# 1-Carb­oxy-3-phenyl­propan-2-aminium chloride

**DOI:** 10.1107/S1600536811041638

**Published:** 2011-10-12

**Authors:** Eric Hosten, Thomas Gerber, Richard Betz

**Affiliations:** a Nelson Mandela Metropolitan University, Summerstrand Campus, Department of Chemistry, University Way, Summerstrand, PO Box 77000, Port Elizabeth 6031, South Africa

## Abstract

The title compound, C_9_H_12_NO_2_^+^·Cl^−^, is the hydro­chloride of an *N*-substituted glycine derivative. The non-H atoms of the alkyl part of the mol­ecule lie nearly in a plane (r.m.s. deviation of all fitted non-H atoms = 0.0142 Å). In the crystal structure, O—H⋯Cl, N—H⋯Cl and C—H⋯O hydrogen bonds involving both O atoms as well as C—H⋯Cl contacts connect the components of the title compound into a three-dimensional network.

## Related literature

For the crystal structure of a palladium coordination compound featuring the ethyl ester of *N*-benzyl­glycine as a ligand, see: Freiesleben *et al.* (1995 ▶). For graph-set analysis of hydrogen bonds, see: Etter *et al.* (1990 ▶); Bernstein *et al.* (1995 ▶).
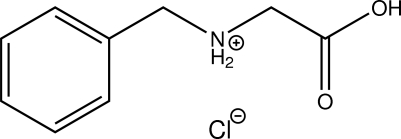


## Experimental

### 

#### Crystal data

C_9_H_12_NO_2_^+^·Cl^−^
*M* *_r_* = 201.65
Orthorhombic, 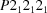

*a* = 5.0290 (7) Å
*b* = 5.4900 (8) Å
*c* = 36.254 (5) Å
*V* = 1000.9 (2) Å^3^
*Z* = 4
Mo *K*α radiation
μ = 0.35 mm^−1^
*T* = 200 K
0.53 × 0.40 × 0.07 mm

#### Data collection

Bruker APEXII CCD diffractometer
Absorption correction: multi-scan (*SADABS*; Bruker, 2008 ▶) *T*_min_ = 0.585, *T*_max_ = 1.000
7535 measured reflections
2376 independent reflections
2273 reflections with *I* > 2σ(*I*)
*R*_int_ = 0.048

#### Refinement

*R*[*F* ^2^ > 2σ(*F* ^2^)] = 0.080
*wR*(*F* ^2^) = 0.177
*S* = 1.34
2376 reflections
125 parameters
H atoms treated by a mixture of independent and constrained refinement
Δρ_max_ = 0.33 e Å^−3^
Δρ_min_ = −0.57 e Å^−3^
Absolute structure: Flack (1983 ▶), 903 Friedel pairs
Flack parameter: 0.1 (3)

### 

Data collection: *APEX2* (Bruker, 2010 ▶); cell refinement: *SAINT* (Bruker, 2010 ▶); data reduction: *SAINT*; program(s) used to solve structure: *SIR97* (Altomare *et al.*, 1999 ▶); program(s) used to refine structure: *SHELXL97* (Sheldrick, 2008 ▶); molecular graphics: *ORTEP-3* (Farrugia, 1997 ▶) and *Mercury* (Macrae *et al.*, 2008 ▶); software used to prepare material for publication: *SHELXL97* and *PLATON* (Spek, 2009 ▶).

## Supplementary Material

Crystal structure: contains datablock(s) I, global. DOI: 10.1107/S1600536811041638/aa2029sup1.cif

Supplementary material file. DOI: 10.1107/S1600536811041638/aa2029Isup2.cdx

Structure factors: contains datablock(s) I. DOI: 10.1107/S1600536811041638/aa2029Isup3.hkl

Supplementary material file. DOI: 10.1107/S1600536811041638/aa2029Isup4.cml

Additional supplementary materials:  crystallographic information; 3D view; checkCIF report

## Figures and Tables

**Table 1:** Hydrogen-bond geometry (Å, °)

*D*—H⋯*A*	*D*—H	H⋯*A*	*D*⋯*A*	*D*—H⋯*A*
O1—H1⋯Cl1^i^	0.84	2.26	3.048 (3)	157
N1—H71⋯Cl1^ii^	0.86 (6)	2.31 (6)	3.166 (5)	178 (6)
N1—H72⋯Cl1	1.07 (6)	2.15 (6)	3.148 (5)	154 (5)
C2—H2*A*⋯O1^iii^	0.99	2.48	3.364 (7)	148
C2—H2*B*⋯O1^iv^	0.99	2.50	3.383 (7)	148
C16—H16⋯Cl1^ii^	0.95	2.78	3.654 (6)	154

Symmetry codes: (i) 
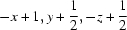
; (ii) 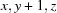; (iii) 
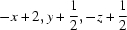
; (iv) 
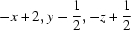
; (v) 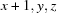.

**Fig. 1.**
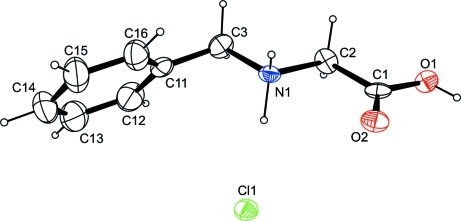
The molecular structure of the title compound, with atom labels and anisotropic displacement ellipsoids (drawn at 50% probability level).

**Fig. 2.**
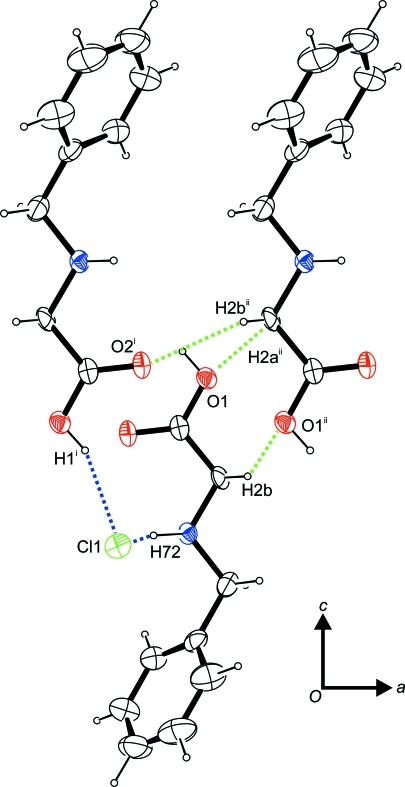
Selected intermolecular contacts, viewed along [0 - 1 0]. Blue dashed lines indicate classical hydrogen bonds, green dashed lines C–H···O contacts. Symmetry operators: i -x + 1, y - 1/2, -z + 1/2; ii -x + 2, y - 1/2, -z + 1/2.

**Fig. 3.**
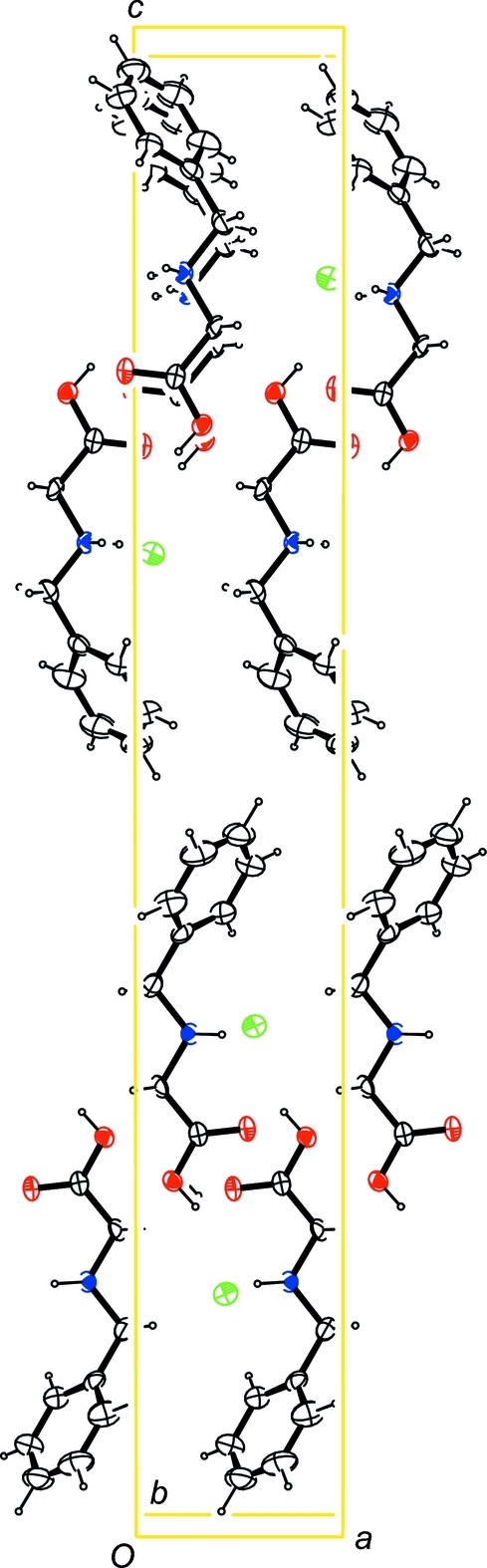
Molecular packing of the title compound, viewed along [0 1 0] (anisotropic displacement ellipsoids drawn at 50% probability level).

## supplementary crystallographic information

### Comment

Amino acids play a major role in the metabolism of living creatures and are characterized by their omnipresence as well as their easy availability in both nature as well as industry. From a chemical viewpoint, their molecular set-up denotes them as potential chelate ligands whose denticity and charge can be influenced by simple variation of the pH value. Coordination compounds featuring amino acids in their ligand sphere might have interesting pharmaceutical properties, especially when keeping in mind that derivatization of the respective amino acids can be used for fine-tuning thermodynamic as well as kinetic characteristics of the compounds and the tailoring of secretion rates on grounds of hydrophilicity. In our continuous efforts in elucidating the rules guiding the formation of *N*,*O*-supported chelate ligands, we investigated the crystal structure of the title compound to enable comparative studies of metrical parameters in envisioned metal complexes. Information about the molecular and crystal structure of a palladium coordination compound featuring the ethyl ester of *N*-benzylglycine is apparent in the literature (Freiesleben *et al.*, 1995).

Intracyclic C–C–C angles cover a range of 118.0 (5)–121.5 (6) ° with the smallest angle found on the substituted carbon atom and the biggest angle in *ortho* position to this atom. The non-hydrogen atoms of the alkyl part of the molecule are nearly in plane (r.m.s of all fitted non-hydrogen atoms = 0.0142 Å). The least-squares planes defined by these atoms on the one hand and the carbon atoms of the aromatic system on the other hand enclose an angle of 60.21 (21) ° (Fig. 1).

In the crystal structure, classical hydrogen bonds as well as C–H···O contacts and C–H···Cl contacts whose range falls by up to more than 0.2 Å below the sum of van-der-Waals radii of the respective atoms are observed. The classical hydrogen bonds are apparent between the nitrogen- and oxygen-bonded hydrogen atoms as donors and – exclusively – the chloride anion as acceptor While the C–H···O contacts are apparent between both hydrogen atoms of the amino acid's methylene group and the oxygen atom of the hydroxyl group, the C–H···Cl contacts stem from one of the aromatic system's hydrogen atoms in *ortho* position to the substituent. In terms of graph-set analysis (Etter *et al.*, 1990; Bernstein *et al.*, 1995), the descriptor for the classical hydrogen bonds is *DDD* on the unitary level while the C–H-supported contacts necessitate a *DC*^1^_1_(4)*C*^1^_1_(4) descriptor on the same level. The C–H···O contacts are present as antidromic chains. In total, the entities of the title compound are connected to a three-dimensional network. π-Stacking is not a prominent feature with the shortest intercentroid distance between two aromatic systems found at 5.029 (4) Å, the length of the *a* axis (Fig. 2).

The packing of the title compound in the crystal is shown in Figure 3.

### Experimental

The compound was obtained commercially (Fluka). Crystals suitable for the X-ray diffraction study were obtained upon slow evaporation of an aqueous solution of the compound at ambient temperature.

### Refinement

Carbon-bound H atoms were placed in calculated positions (C—H 0.95 Å for aromatic C atoms, C—H 0.99 Å for the methylene group) and were included in the refinement in the riding model approximation, with *U*(H) set to 1.2*U*_eq_(C). The H atom of the hydroxyl group was allowed to rotate with a fixed angle around the C—O bond to best fit the experimental electron density (HFIX 147 in the *SHELX* program suite (Sheldrick, 2008)), with *U*(H) set to 1.5*U*_eq_(O). Both nitrogen-bound H atoms were located on a difference Fourier map and refined freely.

### Crystal data

**Table:**

C_9_H_12_NO_2_^+^·Cl^−^	*F*(000) = 424
*M**_r_* = 201.65	*D*_x_ = 1.338 Mg m^−^^3^
Orthorhombic, *P*2_1_2_1_2_1_	Mo *K*α radiation, λ = 0.71069 Å
Hall symbol: P 2ac 2ab	Cell parameters from 7358 reflections
*a* = 5.0290 (7) Å	θ = 2.3–28.5°
*b* = 5.4900 (8) Å	µ = 0.35 mm^−^^1^
*c* = 36.254 (5) Å	*T* = 200 K
*V* = 1000.9 (2) Å^3^	Platelet, colourless
*Z* = 4	0.53 × 0.40 × 0.07 mm

### Data collection

**Table:**

Bruker APEXII CCD diffractometer	2376 independent reflections
Radiation source: fine-focus sealed tube	2273 reflections with *I* > 2σ(*I*)
graphite	*R*_int_ = 0.048
φ and ω scans	θ_max_ = 28.0°, θ_min_ = 2.3°
Absorption correction: multi-scan (*SADABS*; Bruker, 2008)	*h* = −6→6
*T*_min_ = 0.585, *T*_max_ = 1.000	*k* = −7→6
7535 measured reflections	*l* = −47→47

### Refinement

**Table:**

Refinement on *F*^2^	Secondary atom site location: difference Fourier map
Least-squares matrix: full	Hydrogen site location: inferred from neighbouring sites
*R*[*F*^2^ > 2σ(*F*^2^)] = 0.080	H atoms treated by a mixture of independent and constrained refinement
*wR*(*F*^2^) = 0.177	*w* = 1/[σ^2^(*F*_o_^2^) + (0.*P*)^2^ + 3.0055*P*] where *P* = (*F*_o_^2^ + 2*F*_c_^2^)/3
*S* = 1.34	(Δ/σ)_max_ < 0.001
2376 reflections	Δρ_max_ = 0.33 e Å^−^^3^
125 parameters	Δρ_min_ = −0.57 e Å^−^^3^
0 restraints	Absolute structure: Flack (1983), 903 Friedel pairs
Primary atom site location: structure-invariant direct methods	Flack parameter: 0.1 (3)

### Fractional atomic coordinates and isotropic or equivalent isotropic displacement parameters (Å^2^)

**Table:**

	*x*	*y*	*z*	*U*_iso_*/*U*_eq_	
O1	0.8269 (6)	0.7682 (8)	0.26326 (8)	0.0246 (7)	
H1	0.7157	0.7732	0.2805	0.037*	
O2	0.4647 (6)	0.7619 (9)	0.22798 (8)	0.0277 (7)	
N1	0.7400 (7)	0.7563 (9)	0.16416 (10)	0.0223 (7)	
H71	0.661 (12)	0.894 (12)	0.1630 (17)	0.033*	
H72	0.585 (12)	0.623 (10)	0.1641 (16)	0.033*	
C1	0.7017 (8)	0.7659 (11)	0.23163 (12)	0.0225 (8)	
C2	0.8894 (8)	0.7703 (11)	0.19964 (11)	0.0252 (9)	
H2A	0.9953	0.9222	0.2003	0.030*	
H2B	1.0136	0.6308	0.2014	0.030*	
C3	0.9244 (10)	0.7587 (12)	0.13153 (11)	0.0314 (9)	
H3A	1.0672	0.6371	0.1353	0.038*	
H3B	1.0085	0.9212	0.1294	0.038*	
C11	0.7770 (10)	0.7013 (9)	0.09631 (13)	0.0292 (11)	
C12	0.8329 (16)	0.4968 (12)	0.07646 (18)	0.0483 (17)	
H12	0.9657	0.3870	0.0849	0.058*	
C13	0.6936 (18)	0.4473 (14)	0.04329 (18)	0.056 (2)	
H13	0.7350	0.3046	0.0296	0.068*	
C14	0.5016 (16)	0.6007 (14)	0.03072 (17)	0.0542 (19)	
H14	0.4067	0.5654	0.0087	0.065*	
C15	0.4480 (15)	0.8087 (12)	0.05065 (15)	0.0494 (17)	
H15	0.3177	0.9202	0.0420	0.059*	
C16	0.5817 (13)	0.8566 (11)	0.08306 (15)	0.0396 (13)	
H16	0.5390	0.9994	0.0966	0.048*	
Cl1	0.4309 (2)	0.2582 (2)	0.16068 (3)	0.0287 (3)	

### Atomic displacement parameters (Å^2^)

**Table:**

	*U*^11^	*U*^22^	*U*^33^	*U*^12^	*U*^13^	*U*^23^
O1	0.0225 (13)	0.0257 (17)	0.0256 (14)	0.0029 (17)	−0.0031 (11)	0.0055 (18)
O2	0.0164 (13)	0.0295 (16)	0.0370 (16)	−0.0029 (19)	−0.0019 (12)	−0.001 (2)
N1	0.0199 (16)	0.0205 (16)	0.0266 (16)	−0.0063 (19)	−0.0008 (13)	0.000 (2)
C1	0.0207 (18)	0.012 (2)	0.035 (2)	−0.002 (2)	−0.0030 (16)	0.005 (2)
C2	0.0186 (19)	0.027 (2)	0.030 (2)	0.011 (2)	−0.0074 (16)	−0.001 (2)
C3	0.026 (2)	0.035 (2)	0.034 (2)	0.002 (4)	0.0066 (19)	0.002 (3)
C11	0.030 (2)	0.031 (3)	0.027 (2)	−0.008 (2)	0.0090 (19)	0.0008 (19)
C12	0.069 (5)	0.034 (3)	0.042 (3)	0.007 (3)	0.008 (3)	−0.002 (3)
C13	0.082 (6)	0.049 (4)	0.039 (3)	−0.007 (4)	0.012 (4)	−0.013 (3)
C14	0.063 (5)	0.069 (5)	0.031 (3)	−0.019 (4)	−0.002 (3)	−0.010 (3)
C15	0.052 (4)	0.064 (5)	0.032 (3)	−0.002 (4)	−0.007 (3)	−0.003 (3)
C16	0.040 (3)	0.048 (3)	0.031 (3)	−0.001 (3)	0.002 (3)	−0.005 (2)
Cl1	0.0357 (5)	0.0192 (4)	0.0312 (5)	−0.0014 (7)	0.0024 (5)	−0.0005 (6)

### Geometric parameters (Å, °)

**Table:**

O1—C1	1.308 (5)	C3—H3B	0.9900
O1—H1	0.8400	C11—C12	1.363 (8)
O2—C1	1.199 (5)	C11—C16	1.386 (8)
N1—C2	1.492 (5)	C12—C13	1.418 (10)
N1—C3	1.503 (5)	C12—H12	0.9500
N1—H71	0.86 (6)	C13—C14	1.360 (11)
N1—H72	1.07 (6)	C13—H13	0.9500
C1—C2	1.496 (6)	C14—C15	1.378 (9)
C2—H2A	0.9900	C14—H14	0.9500
C2—H2B	0.9900	C15—C16	1.379 (8)
C3—C11	1.510 (6)	C15—H15	0.9500
C3—H3A	0.9900	C16—H16	0.9500
			
C1—O1—H1	109.5	C11—C3—H3B	109.4
C2—N1—C3	111.5 (3)	H3A—C3—H3B	108.0
C2—N1—H71	103 (4)	C12—C11—C16	118.0 (5)
C3—N1—H71	104 (4)	C12—C11—C3	121.1 (5)
C2—N1—H72	114 (3)	C16—C11—C3	120.8 (5)
C3—N1—H72	117 (3)	C11—C12—C13	120.2 (7)
H71—N1—H72	105 (5)	C11—C12—H12	119.9
O2—C1—O1	125.1 (4)	C13—C12—H12	119.9
O2—C1—C2	122.8 (4)	C14—C13—C12	121.1 (7)
O1—C1—C2	112.1 (4)	C14—C13—H13	119.5
N1—C2—C1	110.5 (3)	C12—C13—H13	119.5
N1—C2—H2A	109.6	C13—C14—C15	118.5 (6)
C1—C2—H2A	109.6	C13—C14—H14	120.8
N1—C2—H2B	109.6	C15—C14—H14	120.8
C1—C2—H2B	109.6	C14—C15—C16	120.6 (7)
H2A—C2—H2B	108.1	C14—C15—H15	119.7
N1—C3—C11	111.1 (4)	C16—C15—H15	119.7
N1—C3—H3A	109.4	C15—C16—C11	121.5 (6)
C11—C3—H3A	109.4	C15—C16—H16	119.2
N1—C3—H3B	109.4	C11—C16—H16	119.2
			
C3—N1—C2—C1	−179.6 (5)	C3—C11—C12—C13	−179.5 (6)
O2—C1—C2—N1	−2.8 (9)	C11—C12—C13—C14	−0.4 (11)
O1—C1—C2—N1	177.5 (5)	C12—C13—C14—C15	1.1 (11)
C2—N1—C3—C11	170.2 (5)	C13—C14—C15—C16	−1.6 (10)
N1—C3—C11—C12	−115.9 (6)	C14—C15—C16—C11	1.3 (10)
N1—C3—C11—C16	64.6 (7)	C12—C11—C16—C15	−0.5 (9)
C16—C11—C12—C13	0.0 (9)	C3—C11—C16—C15	179.0 (5)

### Hydrogen-bond geometry (Å, °)

**Table:**

*D*—H···*A*	*D*—H	H···*A*	*D*···*A*	*D*—H···*A*
O1—H1···Cl1^i^	0.84	2.26	3.048 (3)	157.
N1—H71···Cl1^ii^	0.86 (6)	2.31 (6)	3.166 (5)	178 (6)
N1—H72···Cl1	1.07 (6)	2.15 (6)	3.148 (5)	154 (5)
C2—H2A···O1^iii^	0.99	2.48	3.364 (7)	148.
C2—H2B···O1^iv^	0.99	2.50	3.383 (7)	148.
C2—H2B···O2^v^	0.99	2.57	3.070 (5)	111.
C16—H16···Cl1^ii^	0.95	2.78	3.654 (6)	154.

Symmetry codes: (i) −*x*+1, *y*+1/2, −*z*+1/2; (ii) *x*, *y*+1, *z*; (iii) −*x*+2, *y*+1/2, −*z*+1/2; (iv) −*x*+2, *y*−1/2, −*z*+1/2; (v) *x*+1, *y*, *z*.
